# Explanation-Driven Deep Learning Model for Prediction of Brain Tumour Status Using MRI Image Data

**DOI:** 10.3389/fgene.2022.822666

**Published:** 2022-03-14

**Authors:** Loveleen Gaur, Mohan Bhandari, Tanvi Razdan, Saurav Mallik, Zhongming Zhao

**Affiliations:** ^1^ Amity International Business School, Amity University, Noida, India; ^2^ Nepal College of Information Technology, Lalitpur, Nepal; ^3^ Center for Precision Health, School of Biomedical Informatics, The University of Texas Health Science Center at Houston, Houston, TX, United States; ^4^ Human Genetics Center, School of Public Health, The University of Texas Health Science Center at Houston, Houston, TX, United States

**Keywords:** LIME, SHAP, XAI, brain tumor, MRI

## Abstract

Cancer research has seen explosive development exploring deep learning (DL) techniques for analysing magnetic resonance imaging (MRI) images for predicting brain tumours. We have observed a substantial gap in explanation, interpretability, and high accuracy for DL models. Consequently, we propose an explanation-driven DL model by utilising a convolutional neural network (CNN), local interpretable model-agnostic explanation (LIME), and Shapley additive explanation (SHAP) for the prediction of discrete subtypes of brain tumours (meningioma, glioma, and pituitary) using an MRI image dataset. Unlike previous models, our model used a dual-input CNN approach to prevail over the classification challenge with images of inferior quality in terms of noise and metal artifacts by adding Gaussian noise. Our CNN training results reveal 94.64% accuracy as compared to other state-of-the-art methods. We used SHAP to ensure consistency and local accuracy for interpretation as Shapley values examine all future predictions applying all possible combinations of inputs. In contrast, LIME constructs sparse linear models around each prediction to illustrate how the model operates in the immediate area. Our emphasis for this study is interpretability and high accuracy, which is critical for realising disparities in predictive performance, helpful in developing trust, and essential in integration into clinical practice. The proposed method has a vast clinical application that could potentially be used for mass screening in resource-constraint countries.

## 1 Introduction

According to the world health organization (WHO) world cancer report (2020), cancer is amongst the leading death-causing diseases, ranked second (after cardiovascular disease), accounting for nearly 10 million deaths in 2020 (Sung et al., 2021). Compared to other diagnoses, cancer screening is a different and more complicated public health approach that needs extra resources, infrastructure, and coordination. The WHO recommends the implementation of screening programs when the following conditions are fulfilled (Sung et al., 2021):

1. The efficiency of tool/model/software has been demonstrated
2. Sufficient resources and facilities to confirm diagnoses and treatments are available
3. The prevalence of the disease is extreme enough to justify the screening

The total prevalence of all central nervous system tumours is 3.9 per 100,000 persons worldwide; the incidence differs with age, gender, race, and region and is extremely frequent in Northern Europe, followed by Australia, the United States, and Canada. Meningioma is the most common one, accounting for 36.8% of all tumours; glioma is the most widespread malignant tumour, accounting for 75% of central nervous system malignant tumours, with a total incidence of six cases per 100,000 people per year. MRI is presently the ideal method for early detection of human brain tumours as it is non-invasive (Spatharou et al., 2021). However, the interpretation of MRI is predominantly centred on the opinions of radiologists.

The advent of convolution neural network (CNN)-based deep learning (DL) provides the basis for imaging-based artificial intelligence (AI) solutions. DL-guided solutions intend to supplement clinical decision making. There are several motives why the proposed architecture is a CNN-based DL architecture. First, it is observed that CNN-based DL is extremely good at lowering the threshold of parameters while maintaining model quality. Second, it does not require human feature engineering because it can automatically extract features from an image. Third, the literature supports the CNN-based DL model by several researchers and that it has achieved good image classification and recognition accuracy. However, it is crucial to observe that very few researchers have applied local interpretable model-agnostic explanation (LIME) and Shapley additive explanation (SHAP) along with CNN. Researchers demonstrated the immense potential of imaging tools to mitigate the heavy burden on medical experts (Wojciech et al., 2017). It further allows devoting additional help in patient care, reducing burnout, and shrinking overall medical costs for patients (Dave et al., 2020). Working on the detection system, Gupta et al. (2016) applied DL algorithms, Resnet50, to distinguish COVID-19 from X-rays to achieve a fully autonomous and speedier diagnosis. With an average COVID-19 detection time of roughly 2.5 s and an average accuracy of 0.97, the authors aimed to minimise the run time to about 2.5 s. Kollias et al. (2018) introduced different performance indicators such as precision, responsiveness, specificity, precision, F1 value, and DL. The results showed a standard accuracy of 92.93% and sensitivity of 94.79% to provide robust identification and detection of COVID-19 in the chest X-ray dataset. In one of the research (Ke et al., 2019), the deep neural network correlation learning mechanism for CT brain tumour detection used palettes of CNN architecture to adjust them to the best possible detection result of ANN. The AISA framework for MRI data analysis demonstrated its application to brain scan data by deriving independent subspaces and extracting texture features. Then, dimensionality is reduced using t-SNE embedding for discriminative classification. Finally, the KNN classification is applied. Despite the immense popularity of DL models in clinical decision making, the lack of interpretability and transparency by algorithm-driven decisions remains the biggest challenge, particularly in medical settings. Although, many researchers (Richard et al., 2020; Zucco et al., 2018) observed various impediments in developing XAI-based clinical decision support systems (CDSS) due to the non-availability of any universal notion of explainability. Our study proposes an explanation-driven DL-based model to predict distinctive subtypes of brain tumours (meningioma, glioma, and pituitary) using an MRI image dataset. We also implemented LIME and Shapley additive explanations to create more transparency in the models while keeping intact a high performance rate. Our study will help the users (medical professionals, clinicians, etc.) in comprehending and efficiently managing the ever-increasing number of trustable and reliable AI partners (Sharma et al., 2020).

Compared to previous models, our model used a dual-input CNN approach to prevail over the classification challenge with inferior-quality images and an accuracy of 94.64% compared to other state-of-the-art models. Previous studies lack explanation, and thus, we used Explainable AI (XAI) algorithms such as LIME and SHAP, which is the differentiating element of this study. We used SHAP to ensure consistency and local accuracy for interpretation as Shapley values examine all potential predictions using all possible combinations of inputs. Conversely, LIME constructs sparse linear models around each prediction to describe how the model operates in the immediate area.

The deep neural network correlation learning mechanism for computed tomography (CT) brain tumour detection used palettes of CNN architecture to adjust them to the best possible detection result of DL. Though the previously suggested models have higher accuracy, they lack explainability, interpretability, and transparency (Abdalla and Esmail, 2018; Khairandish et al., 2021). The proposed model used XAI algorithms such as LIME (Vedaldi and Soatto, 2008) and SHAP as detailed in Algorithm 2.

The contributions in this study are summarised in what follows:

1. We aimed to create an explanation-driven multi-input DL model where SHAP and LIME are used for an in-depth description of results. One set of two input datasets is fed to the convolution layer and one to the fully connected layer.
2. We have achieved high accuracy of (94.64%) brain MRI images compared to other state-of-the-art models.

## 2 Methods

### 2.1 Datasets

In this study, we used the publicly available MRI images (Bhuvaji, 2020). The datasets are annotated into three categories of tumours: glioma tumour, meningioma tumour, and pituitary tumour, along with the normal image. Out of 2,870 total images, 2,296 images of distinct types are used as training sets and the remaining as test sets.

#### 2.1.1 Data Pre-Processing

**FIGURE 1:**
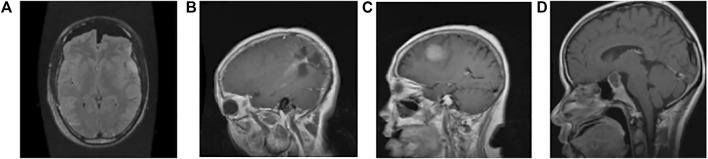
Sample image data of different types of tumours. **(A)** Normal: the intensity of the parenchyma in the brain without any tumour is normal. The ventricular system and cisternal spaces are supposed to be in good working order. There is always no evidence of an intracranial space-occupying lesion (Gaillard, 2021). **(B)** Glioma tumour: gliomas have thick, irregularly enhancing borders of the focal necrotic core with a haemorrhagic component. They are surrounded by vasogenic-type oedema, containing malignant cell infiltration. Intratumoural haemorrhage happens rarely (less than 2%) (Frank, 2021) **(C)** Meningioma tumour: meningiomas are extra-axial tumours arising from meningocytes or arachnoid cap cells of meninges and can be found where meninges exist, as well as in some sites where only rest cells are thought to exist (Gaillard and Rasuli, 2021) **(D)** Pituitary tumour: for pituitary adenomas, minor intra-pituitary lesions appear differently than larger lesions that spread into the suprasellar region and pose various surgical and diagnostic issues. Based on tumour aspects, overall signal qualities can vary (Weerakkody and Gaillard, 2021).

All 512 × 512 × 3 images are resized to 150 × 150 × 3. The images are rearranged for faster convergence and preventing the CNN model from learning the training order. For better classification results, we have introduced Gaussian noise as it improves the learning for DL (Neelakantan et al., 2015) with mean = 0 and standard deviation 10^0.5^. Figure 1 shows a single instance among the categories of tumours from the dataset.

### 2.2 Proposed Framework

**FIGURE 2:**
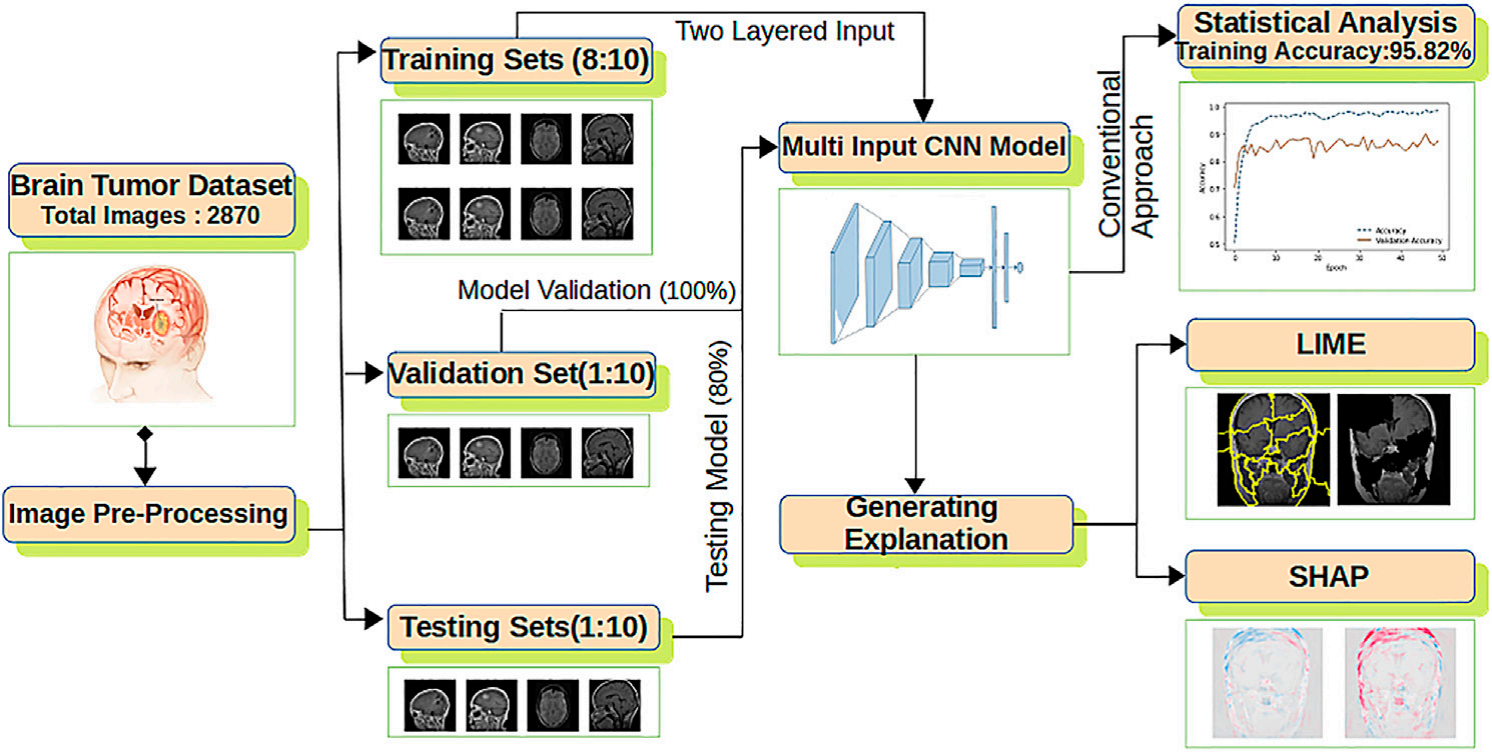
The proposed explanation-driven DL model for prediction of brain tumour status using MRI image data: 2870 MRI images are pre-processed and divided into training, validation, and test sets. Two copies of datasets are fed into a multi-input CNN model to find the training, validation, and test accuracy. The same CNN model was further imposed on LIME and SHAP.

The overall architecture of the model used is shown in Figure 2 composed of feature extraction, a CNN model, statistical performance measures, and explanation extraction frameworks.

For improved accuracy, two copies of the dataset are fed to the CNN model having an output layer of size 1 × 4 and six hidden layers (Yu et al., 2017). Adam optimiser with its default parameters is applied with the rectified linear unit (ReLU) and softmax as the activation function. The final CNN model is used for statistical accuracy measurement, LIME and SHAP. For LIME explanations, perturbation is calculated, whereas for SHAP, a gradient explainer is applied. The whole process is formalized in Algorithm 1.

Algorithm 1 Explanation-driven multi-input DL model for prediction of brain tumour.

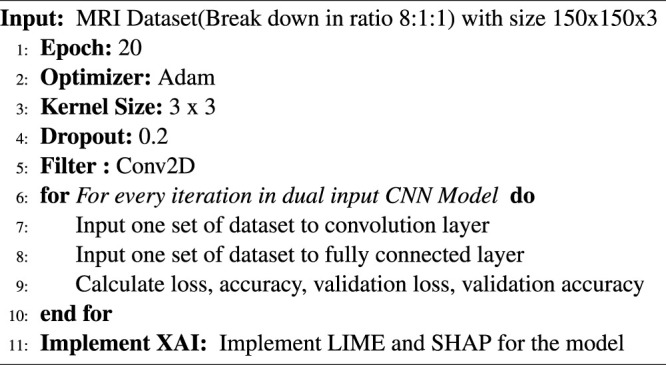

For the classification task in the proposed explainable model, a CNN with dual-input architecture is used. The CNN is imposed with ReLU as activation in all hidden layers. Compared with the input value and zero value, ReLU is simple to calculate. Furthermore, ReLU has a derivative of either 0 or 1 based on positive or negative input. This feature of ReLU is essential in comparing explainable modules such as LIME and SHAP. Adam optimiser with its default parameter (Kingma and Ba, 2015) is used along with sparse categorical cross entropy; the kernel size is set to 3 × 3.

## 3 Results

**FIGURE 3:**
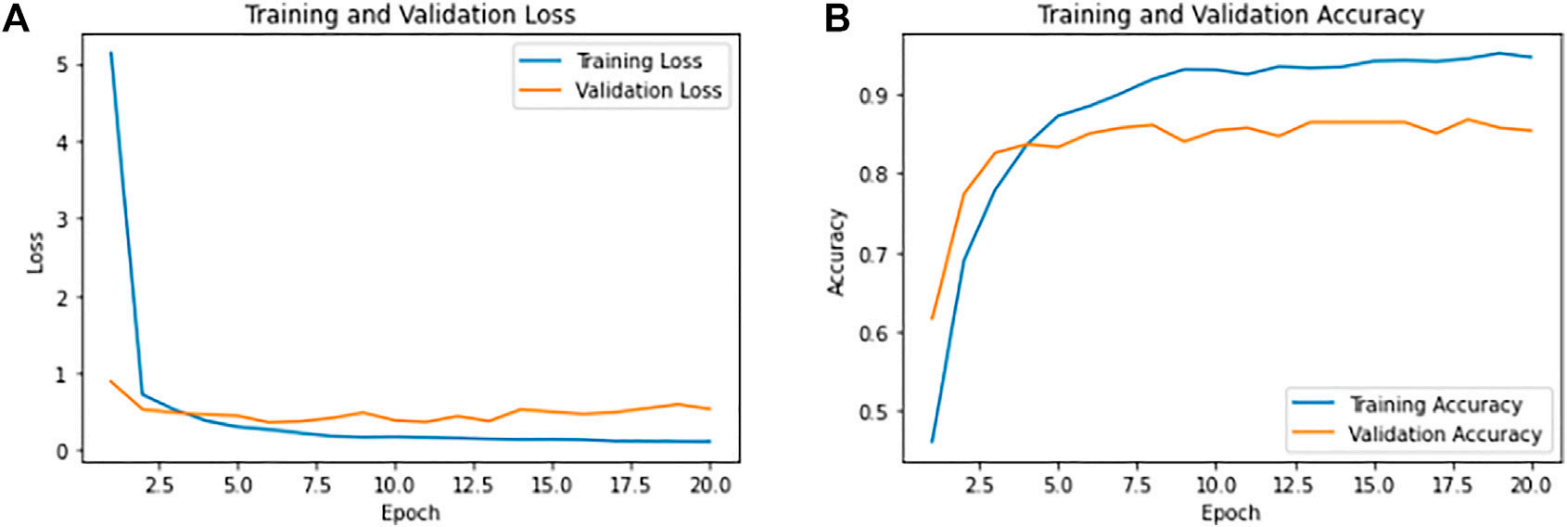
Training and validation results of CNN. **(A)** Shows the overall training loss as 0.1149 and validation loss as 0.53. **(B)** Shows the overall training accuracy as 94.64% and validation accuracy of 85.37%.

Following the classification process, the performance of CNN models is evaluated based on accuracy and the number of wrong predictions. The curves for the conventional results of CNN are presented in Figure 3.

### 3.1 CNN

The model was iterated for 20 epochs, and during callback in CNN modules, we had monitored the loss with min mode and patience level of three to cross the over-fitting. Achieving the training accuracy of 94.64% and overall test accuracy of 85.37%, the model has 26 wrong predictions with 0.1149 as training loss and 0.53 as validation loss.

Furthermore, to estimate the performance of the CNN model on the configured dataset, K-fold cross validation is performed with K = 10 non-overlapping folds for 20 epochs with a batch size of 128. The test and train sets were split in the ratio of 1:4. The final validation result of the cross fold is shown in Table 1. The proposed model has achieved almost 100% training accuracy during cross validation.

**TABLE 1:** K-fold cross-validation results.

Fold	Final validation loss	Final validation accuracy (%)
1	0.011 44	99.5
2	0.017 06	98.47
3	0.021 52	99.13
4	0.009 88	99.34
5	0.005 54	100
6	0.012 98	99.13
7	0.008 74	99.78
8	0.005 33	99.78
9	0.010 18	99.34
10	0.008 8	99.56

Table 2 shows the confusion matrix for 287 test images. A total of 7 normal images out of 46, 14 glioma images out of 84, 12 meningioma images out of 77, and 3 pituitary images out of 80 were misclassified.

**TABLE 2:** Confusion matrix for the CNN.

		Actual value			
		Normal	Glioma	Meningioma	Pituitary
Predicted values	Normal	37	8	1	0
	Glioma	7	70	5	2
	Meningioma	0	12	65	0
	Pituitary	0	3	0	77

To validate our model statistically, we performed McNemar’s test (Smith et al., 2020). For labels of test data and labels of model prediction under test data, McNemar’s test gave a chi-squared value of 42.022 and *p* value 9.02 x e^−11^. We can reject the null-hypothesis that both labels perform equally well on the test set, since the *p* value is smaller than *α* = 0.005.

### 3.2 SHAP

For each pixel on a predicted image, the scores show its contribution and can be used to explain tumour classification tasks. The Shapley values correspond to each feature for different categories of the tumour according to Algorithm 2.

Algorithm 2 Algorithm to calculate the Shapley values.

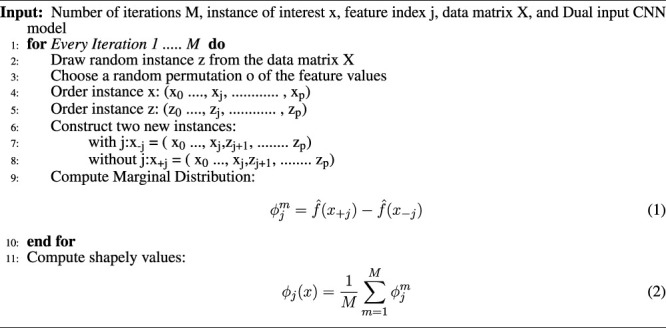

**FIGURE 4:**
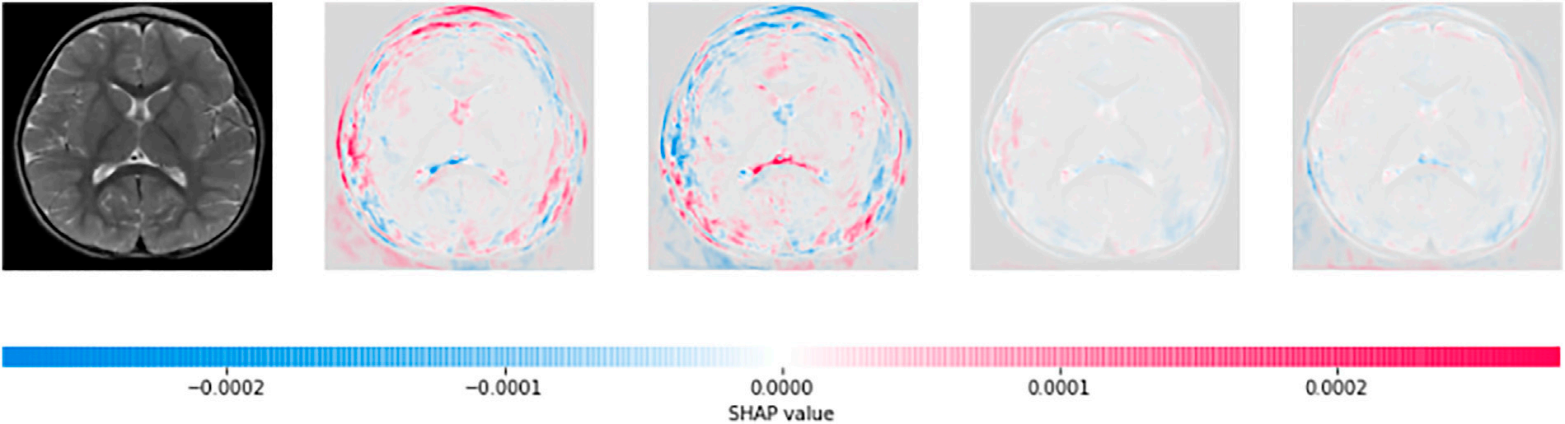
On the basis of Shapley values, we can say that the MRI image is normal.

**FIGURE 5:**
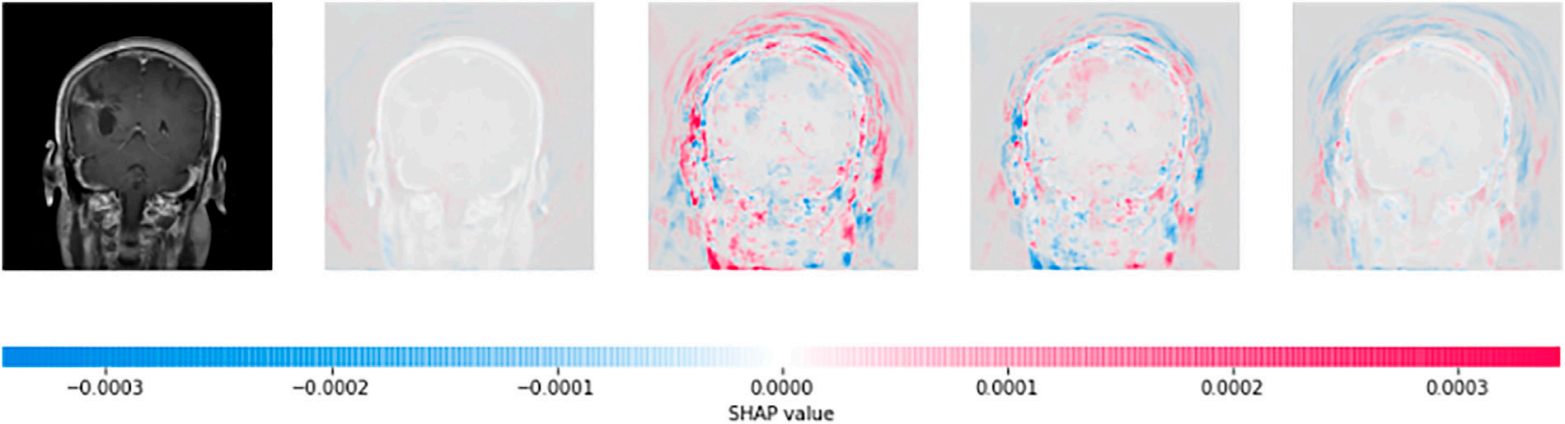
On the basis of Shapley values, we can say that the MRI image holds meningioma tumour.

The CNN model with mathematical behaviour is complicated to interpret directly. Thus, the effect of individual input features on the model’s output is clearly explained using SHAP and shown in Figures 4, 5. Positive SHAP values that raise the likelihood of the class are represented by red pixels. In contrast, negative SHAP values that lower the probability of the class are represented by blue pixels. Figure 4 and Figure 5 are test images. In contrast, the rest of the figures indicate the normal image and three other categories of tumour: glioma, meningioma, and pituitary tumours in successive order.

### 3.3 LIME

**FIGURE 6:**
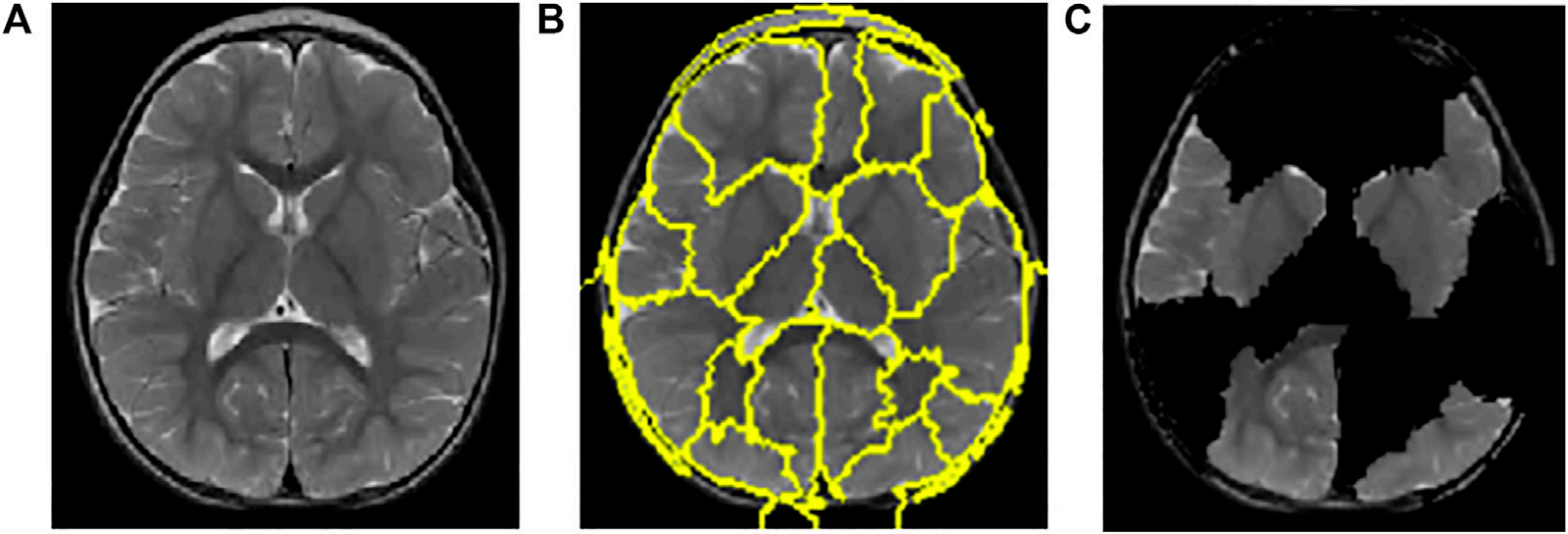
Interpretations generated by LIME for a normal image. **(A)** Sample of the normal image from the test image. **(B)** Superpixels generated from a sample of the normal image from test image quick-shift segmentation to create perturbations. **(C)** Final perturbed image for the normal image.

**FIGURE 7:**
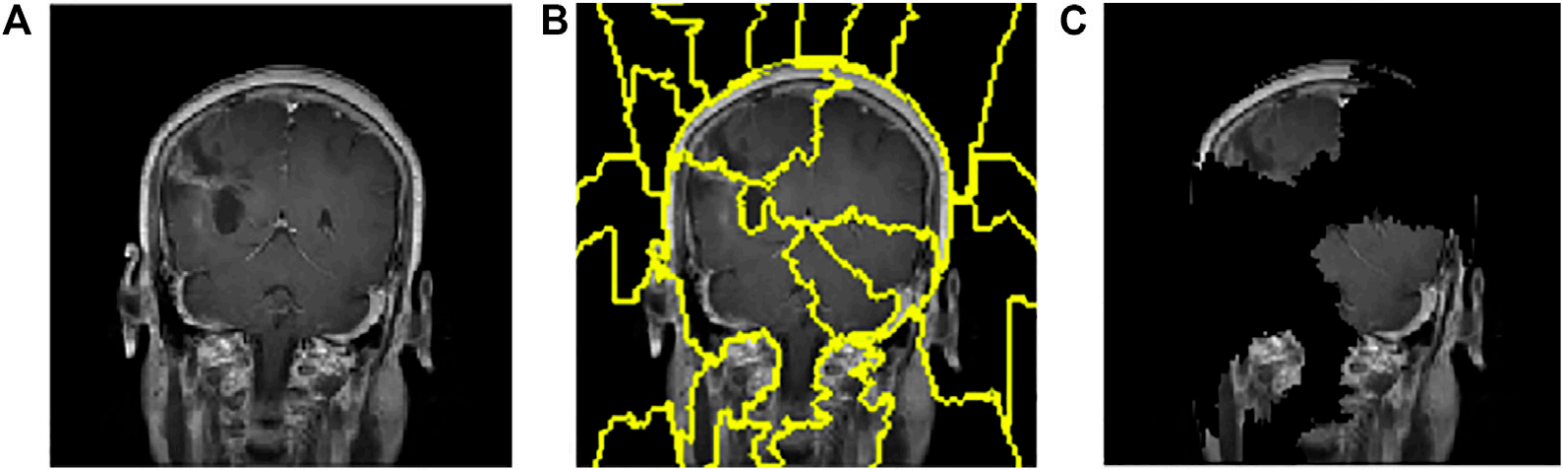
Interpretations generated by LIME for meningioma tumour. **(A)** Sample of meningioma tumour from the test image. **(B)** Superpixels generated from quick-shift segmentation to create perturbations. **(C)** Final perturbed image showing meningioma tumour.

A total of 150 perturbations are used. Random ones and zeros are produced and formed into a matrix, with perturbations as rows and superpixels as columns. A superpixel is ON if it is 1, and it is OFF if it is 0. The length of the displayed vector represents the number of superpixels in the image. The test image is perturbed based on the perturbation vector and predefined superpixels (Vedaldi and Soatto, 2008). The final perturbed image is shown in Figure 6C for normal test image under consideration and in Figure 7C for test image under consideration with meningioma tumour, which shows the portion of the image having a major role for classification.

The CNN model is utilised to generate the explanation using LIME. Figure 6Ais a normal image, and Figure 7A is under the meningioma category. The classification produces a vector of 2,870 probabilities for each category accessible in the CNN model. The quick-shift segmentation method is used to create superpixels. 22 superpixels are generated for Figure 6A and shown in Figure 6B, and 24 superpixels are calculated for Figure 7A and shown in Figure 7B.

## 4 Discussion

### 4.1 Comparison of the Proposed Feature Extraction Methods Using Traditional Machine learning (ML) Methods

We compare the proposed feature extraction methods to traditional ML methods. The comparative results are presented in Table 3. Minz and Mahobiya (2017) pre-processed the MICCAI BraTS dataset to eliminate noise and employed the GLCM (gray-level co-occurrence matrix) for feature extraction and classification boosting (Adaboost). An MRI was used to extract 22 characteristics. The Adaboost classifier is utilised for classification, and the suggested system achieves a maximum accuracy of 89.90%. Abdalla and Esmail (2018) executed a computer-aided detection system after collecting the MRI images. They processed the image before implementing the back-propagation algorithm and extracted the features using Haralick’s features based on the spatial gray-level dependency matrix (SGLD). The results were 99%, but the study could not focus on the explainable section in the training images. A comparative study between support vector machine (SVM) and random forest (RF) classified benign and malignant tumours. First, the brain tumour’s region of interest was determined for feature extraction, and then, features were calculated. Shape characteristics were obtained and utilised to classify benign and malignant tumours. According to the authors, RF (81.90%) outperformed the SVM (78.57%). By combining principal component analysis (PCA), KSVM, and GRB kernels, Arora and Ratan (2021) established a unique technique for categorisation of MRI brain images using discrete wavelet transform (DWT). The experiment was carried out with four different kernels. The findings demonstrate that combining DWT, PCA, KSVM, and the GRB kernel yields the highest accuracy compared to other methodologies. The results show that the time it takes to classify a segmented picture significantly decreases, which might be a watershed moment in the medical profession for tumour diagnosis. Martinez et al. (2020) worked on the FLAIR images on the BRATS 2015 training dataset; it is used to restructure and increase data attributes that lead to a pixel-based classifier. The U-net suggested method performs a semantic segmentation with a precision of 76%, which increases by 23% compared to the random forest classifier with synthetic minority oversampling technique (SMOTE) class balancing algorithm.

**TABLE 3:** Brain tumour detection using traditional ML methods.

Authors	Algorithm	Dataset	Accuracy (%)	XAI
Martinez et al. (2020)	Random Forest	BraTs Dataset	76	No
Minz and Mahobiya (2017)	Adaboost Classifier	BraTs Dataset	89.90	No
Abdalla and Esmail (2018)	Back-Propagation Network	MRI Images	99	No
Asodekar and Gore (2019)	Random Forest	BraTs Dataset	81.90	No
Asodekar and Gore (2019)	SVM	BraTs Dataset	78.57	No
Proposed model	Dual-Input CNN	MRI Images	94.64	Yes

### 4.2 Comparison of the Proposed Method With the Other State-of-the-Art Methods

This section compares our dual-input CNN model with other state-of-the-art models. The results are compared in Table 4. After several data-collection and pre-processing steps such as average filtering segmentation, the DL model was implemented by researchers (Hemanth et al., 2019). In comparison to existing approaches such as conditional random field (89%), SVM (84.5%), and genetic algorithm (GA) (83.64%), the research represents overall performance and comparative output on the brain MRI images. In contrast to existing algorithms, the suggested CNN (91%) produces improved results. The TensorFlow library was used to construct a DL method called faster R-CNN in the work of Avsar and Salcin (2019), and the classifier algorithm was trained and tested using a publicly available dataset of 3,064 MRI brain pictures (708 meningiomas, 1,426 gliomas, and 930 pituitary gland tumours) from 233 patients. The quicker RCNN algorithm has been demonstrated to attain 91.66% accuracy, which is exceptional compared to past work on the same dataset. Ranjbarzadeh et al. (2021) proposed a cascaded convolutional neural network (C-ConvNet/C-CNN). A simple but effective cascade, the CNN model, has been suggested to extract local and global characteristics in two methods, with different extraction patches in each. Those patches were chosen to feed the network that their centre was located inside this area after extracting the tumour’s predicted location using a sophisticated pre-processing strategy. As a result of removing a high number of insignificant pixels from the picture in the pre-processing stage, the computing time and ability to generate quick predictions for categorising the clinical image are reduced. The results were compared to other algorithms. Still, the CNN model achieved the highest accuracy (92.03%) on the whole Dice score (mean) and the highest precision (97.12%) on the core sensitivity score (mean). Khairandish et al. (2021) made use of a hybrid model of CNN and SVM in phrases of classification, type, and threshold-based segmentation in terms of detection to classify benign and malignant tumours in brain MRI images. This hybrid CNN–SVM is rated as having an overall accuracy of 98.49%. Still, their study does not show evidence for manipulating low-quality images and XAI. Shahzadi et al. (2018) proposed a CNN cascade with a long short-term memory (LSTM) network for classifying 3D brain tumour MRIs into HG and LG glioma. The features from the pre-trained VGG-16 were retrieved and fed into an LSTM network for learning high-level feature representations. The components extracted from VGG-16 had a classification accuracy of 84%, higher than that of those extracted from AlexNet and ResNet, 71%. Isola et al. (2018) investigated conditional adversarial networks as a general-purpose solution for image-to-image translation challenges by using a 1,616 PatchGAN. The PatchGAN 70 × 70 reduces these distortions and improves scores slightly. It is observed that scaling to the full 286 × 286 ImageGAN does not significantly improve the visual quality of the findings and results in a considerably lower FCN-score, indicating that conditional adversarial networks are a promising option for many image-to-image translation tasks, especially those involving highly structured graphical outputs. Milletari et al. (2016) proposed an approach to 3D image segmentation based on a volumetric, fully convolutional neural network. The CNN is trained end-to-end on MRI volumes depicting the prostate and predicts segmentation for the whole volume at once. The training was performed on 50 MRI volumes, and the relative manual ground truth annotation was obtained from the PROMISE2012 challenge dataset. The novel objective function was to optimise during training based on the dice overlap coefficient between the predicted segmentation and the ground truth annotation. Han et al. (2020) proposed an unsupervised medical anomaly detection generative adversarial network (MADGAN). This two-step method uses GAN-based multiple adjacent brain MRI slice reconstruction to detect brain anomalies at various stages on multi-sequence structural MRI. MADGAN can detect anomaly on T1 scans at a very early stage, mild cognitive impairment (MCI), with area under the curve (AUC) 0.727, and anomaly detection (AD) at a late stage with AUC 0.894, while detecting brain metastases on T1c scans with AUC 0.921. On multi-sequence MRI, the model may accurately detect the accumulation of subtle anatomical abnormalities and hyper-intense enhancing lesions, such as (particularly late stage) AD and brain metastases, as the first unsupervised varied disease diagnosis. Baur et al. (2020) presented a novel method towards unsupervised AD in brain MRI by embedding the modelling of healthy anatomy into a CycleGAN-based style-transfer task, which is trained to translate healthy brain MRI images to a simulated distribution with lower entropy and vice versa. By filtering high-frequency, low-amplitude signals from lower entropy samples during training, the resulting model suppresses anomalies in reconstructing the input data at test time. The method outperforms the state-of-the-art method in various measures and can deal with high-resolution data, a current pitfall of autoencoder (AE)-based methods. Castiglioni et al. (2021) concentrated on the issues that must be addressed to create AI applications as clinical decision support systems in a real-world setting. A narrative review with a critical appraisal of publications published between 1989 and 2021 was conducted. According to the study, biomedical and healthcare systems are among the most significant domains for AI applications, with medical imaging being the most suited and promising domain. Clarification of specific challenging points facilitates the development of such systems and their translation to clinical practice. Barragán-Montero et al. (2021) showcased the technological pillars of AI, as well as the state-of-the-art methods and their implementation to medical imaging. This review offered an overview of AI, emphasising medical imaging analysis demonstrating the potential of the state-of-the-art ML and DL algorithms to automate and enhance several aspects of clinical practice.

**TABLE 4:** Brain tumour detection using other state-of-the-art models.

Authors	Algorithm	Dataset	Accuracy (%)	XAI
Shahzadi et al. (2018)	CNN with LSTM	MRI Images	84	No
Hemanth et al. (2019)	CNN	MRI Images	91	No
Avsar and Salcin (2019)	R-CNN	MRI Images	91.66	No
Ranjbarzadeh et al. (2021)	C-CNN	BraTs Dataset	92.03	No
Khairandish et al. (2021)	CNN–SVM	MRI Images	98.49	No
Proposed model	Dual-Input CNN	MRI Images	94.69	Yes

## 5 Conclusion and Future Direction

Using an explanation-driven dual-input CNN model for finding if a particular MRI image is subjected to a tumour or not, the proposed study achieved an accuracy of 94.64%. A brain MRI image dataset is used to train and test the proposed CNN model, and the same model was further imposed to SHAP and LIME algorithms for an explanation. Our experiment utilised two dataset copies as input for better feature extraction, one in the convolution layer and another in the fully connected layer. However, any attempt to remove any features decreased the prediction model’s overall performance; hence, no augmentation was carried out. The proposed model is a locally interpreted model with a model-agnostic explanation, shapely explained to describe the results for ordinary people more qualitatively.

In future, classification algorithms with higher accuracy and better optimiser can be used and imposed on XAI. For better clinical issues, the research may be replicated and applied to other XAI algorithms such as GradCAM. Furthermore, like the most recent advances on computing capacity, neuroimaging technologies, and digital phenotyping tools (Ressler and Williams, 2020), algorithms to imitate natural occurrences can be used on heterogeneous datasets for medical imaging modalities, electronic health record engines, multi-omics studies, and real-time monitoring (Rundo et al., 2019).

## Data Availability

Publicly available datasets were analyzed in this study. These data can be found here at https://www.kaggle.com/sartajbhuvaji/brain-tumor-classification-mri.
